# Research Progress of Platinum-Based Complexes in Lung Cancer Treatment: Mechanisms, Applications, and Challenges

**DOI:** 10.3390/ijms26167958

**Published:** 2025-08-18

**Authors:** Wanqi Liang, Yufeng Huang, Yi Wang, Desheng Lu, Qi Sun

**Affiliations:** 1 Department of Pharmacology, International Cancer Center, Shenzhen University Medical School, Shenzhen 518060, China; 2023221088@email.szu.edu.cn (W.L.); 2023224038@email.szu.edu.cn (Y.H.); 2 Shenzhen Key Lab of Functional Polymer, College of Chemistry and Environmental Engineering, Shenzhen University, Shenzhen 518060, China; wangyi0435@szu.edu.cn

**Keywords:** platinum-based complexes, cisplatin, small cell lung cancer, non-small cell lung cancer

## Abstract

Platinum-based complexes, as one of the mainstay chemotherapeutic agents in oncology, have evolved from first-generation Cisplatin to third-generation Oxaliplatin, with numerous platinum complexes currently under clinical investigation. This review systematically evaluates all existing platinum complexes for lung cancer treatment, including three generations of approved agents and investigational candidates, with comprehensive analysis of therapeutic efficacy and toxicity profiles. Our assessment aims to provide novel insights for developing next-generation platinum complexes with optimal therapeutic potential.

## 1. Introduction

Lung cancer is a malignant tumor originating from the bronchial mucosal epithelium or alveolar epithelium. Data from the GLOBOCAN 2022 report released by the International Agency for Research on Cancer (IARC) show that in 2022, there were 1.817 million deaths from lung cancer globally, accounting for 18.7% of all cancer deaths. It is the cancer with the highest incidence and mortality worldwide. The occurrence of lung cancer is associated with multiple genetic mutations and epigenetic changes. Smoking is generally considered the most significant risk factor for lung cancer. With the increasing number of female smokers, the male-to-female ratio has risen from 4:1 to 1.5:1. Based on the type of cancer cells, lung cancer can be divided into small cell lung cancer (SCLC) and non-small cell lung cancer (NSCLC), with NSCLC accounting for 85% of all lung cancers and SCLC accounting for 15%. Common genetic mutations in NSCLC mainly include EGFR, KRAS, EML4-ALK, and ROS1.

Surgical resection is a treatment option for early-stage lung cancer. In 1895, Macewen performed the world’s first total pneumonectomy. In 1933, Dr. Graham from the Washington University School of Medicine in the United States successfully carried out a left total pneumonectomy, and the patient made a full recovery and remained free of lung cancer recurrence until his death in 1963. This marked the official debut of surgical treatment for lung cancer. Subsequently, palliative and radical radiotherapy were successively applied to the treatment of lung cancer.

In 1969, after Barnett Rosenberg’s team confirmed the anticancer activity of Cisplatin, Cisplatin was widely studied and applied to the chemotherapy of solid tumors such as lung cancer, testicular cancer, ovarian cancer, and cervical cancer. The antitumor activity of Cisplatin mainly relies on its covalent binding with DNA purine bases (such as the N7 site of guanine), forming intrastrand or interstrand cross-links, which hinder DNA replication and transcription. It also activates multiple pathways such as p53-dependent apoptosis and oxidative stress, causing tumor cell apoptosis. However, due to the systemic tissue toxicity and drug resistance that occur in the treatment of cancer with Cisplatin, its extensive, long-term, and high-dose use is limited. Therefore, researchers have successively developed second-generation Carboplatin and third-generation Oxaliplatin, as well as other platinum complexes, to reduce some toxicity and drug resistance problems. Currently, the combination of platinum complexes with other drugs (such as paclitaxel and gemcitabine) is the first-line treatment for advanced NSCLC. In recent years, the combination of platinum complexes with immune checkpoint inhibitors has gradually become a research hotspot, such as the combination of Carboplatin/paclitaxel plus pembrolizumab and Cisplatin/gemcitabine plus atezolizumab. It can be seen that platinum complexes occupy an irreplaceable and important position in the treatment of lung cancer. This article comprehensively introduces and evaluates the efficacy of marketed platinum drugs and platinum complexes still in the research stage in the treatment of lung cancer, aiming to identify platinum complexes with potential in lung cancer treatment and to point the way for new directions in lung cancer chemotherapy.

## 2. Results

We have collected most of the information on platinum-based metal complexes for lung cancer in recent years, classified them into four categories—basic drugs, clinical drugs, research drugs, and innovations in targeted drug delivery systems—and presented them below (Table 1).

### 2.1. Platinum-Based Complexes

#### 2.1.1. First Generation: Cisplatin

**Figure 1:**
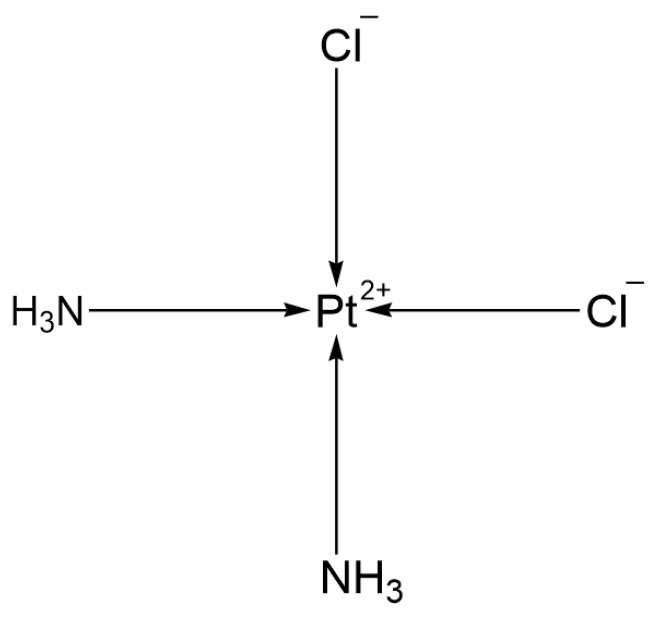
The structure of Cisplatin.

Cisplatin (DDP, Figure 1) was first approved by the US Food and Drug Administration (FDA) in December 1978 for the clinical treatment of testicular cancer. Its applications have since expanded, including the treatment of lung cancer. After Cisplatin enters the cell, its chloride ions are replaced by water molecules, forming a hydrate. This hydrate can bind to the N7 site of the purine bases of DNA, forming DNA-platinum cross-links, which interfere with the replication and transcription of DNA, leading to cell cycle arrest and ultimately inducing cell death. In addition, Cisplatin can also target mitochondria to induce oxidative stress, uncouple oxidative phosphorylation, cause calcium efflux from mitochondria, and activate the caspase-mediated apoptosis pathway, among other mechanisms to kill tumor cells [1]. Cisplatin can enhance the cytotoxic effects of radiation. When used in combination with radiotherapy, it significantly improves the survival rate (SR) of patients with NSCLC. For instance, the 1-year, 2-year, and 3-year SRs for the group receiving daily Cisplatin combined with radiotherapy are 54%, 26%, and 16%, respectively, compared to 46%, 13%, and 2% for the group receiving radiotherapy alone [2]. However, the clinical application of Cisplatin is limited by two major bottlenecks: systemic toxicity and drug resistance issues.

Cisplatin is highly prone to causing nephrotoxicity and associated issues. A clinical study on Cisplatin showed that in 80 cases of treating NSCLC patients with pleural effusion using low-dose Cisplatin, the overall response rate (ORR) reached 83%, effectively inhibiting the occurrence of malignant pleuritis. However, this treatment regimen also triggered a variety of non-hematologic toxic reactions, including nausea (4%), vomiting (3%), empyema (1%), and dyspnea (1%) [3]. Chemotherapy regimens based on Cisplatin often lead to more frequent grade 3 or 4 nausea, vomiting, and nephrotoxicity [4].

Cisplatin resistance is characterized by the interaction of multiple pathways. The autophagy system forms autophagosomes that engulf Cisplatin or drug carriers (such as liposomes) and transport them to lysosomes for degradation or expulsion from the cell, resulting in a decrease in intracellular Cisplatin accumulation. In addition, tumor cells can promote their own invasion and metastasis through epithelial-mesenchymal transition (EMT), thereby leading to drug resistance. In drug-resistant cells, miR-448 and miR-495, respectively, target and regulate the overexpression of multidrug resistance protein 1 (MDR1) and copper-transporting P-type adenosine triphosphatases ATP7A, leading to increased drug efflux. Moreover, the downregulation of the high-affinity copper transporter Ctr1 reduces cellular platinum uptake and decreases intracellular platinum accumulation in drug-resistant cells [5,6].

#### 2.1.2. Second Generation: Carboplatin

**Figure 2:**
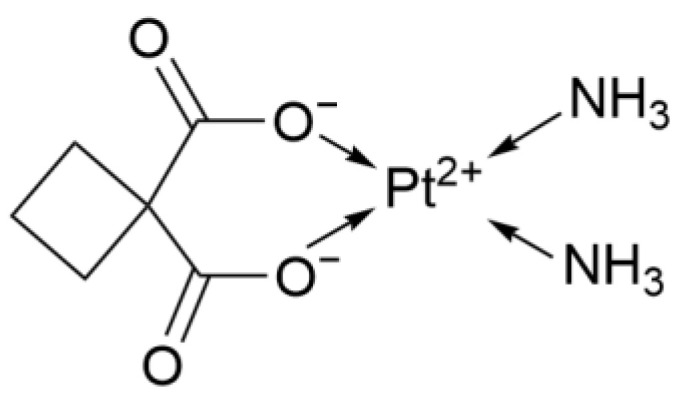
The structure of Carboplatin.

Carboplatin (CBP, Figure 2), whose anticancer activity was discovered by Michael J. Cleare and others in 1980, was first launched in the UK in 1986. Carboplatin introduced the cyclobutanedicarboxylate ligand to replace chloride ions, which increased the molecule’s polarity and thereby enhanced its solubility in water. Moreover, the two carboxyl groups form a stable chelation ring with the platinum atom.

Compared with its predecessor, Carboplatin has two major advantages. First, Carboplatin is 16 times more soluble in water than Cisplatin and has better chemical stability. Therefore, its reactivity with DNA and proteins is lower. The lower protein binding rate allows it to be more easily excreted from the body, thereby reducing ototoxicity and nephrotoxicity. Second, Cisplatin has greater toxicity to rapidly proliferating cells, such as gastrointestinal mucosal cells, which can lead to severe gastrointestinal reactions. In contrast, Carboplatin has lower toxicity and relatively milder gastrointestinal reactions.

However, due to its lower plasma protein binding rate (approximately 24%) and the reversible nature of its binding to plasma proteins, the free form of Carboplatin is more likely to bind with bone marrow cells, leading to bone marrow suppression. Additionally, because Carboplatin has a longer ultrafiltrable platinum half-life, meaning it stays in the body for a longer period, its inhibitory effect on bone marrow cells is more prolonged.

In clinical studies on lung cancer, multiple datasets have shown that patients with advanced NSCLC treated with Carboplatin or Cisplatin in combination with third-generation chemotherapeutic drugs demonstrated comparable overall survival (OS), one-year OS, and ORR. [7]. In terms of adverse events, Cisplatin-based chemotherapy is more likely to cause frequent Grade 3 or 4 nausea and vomiting, as well as nephrotoxicity and ototoxicity, whereas Carboplatin is more inclined to cause hematologic toxicities, such as thrombocytopenia and leukopenia. Although Cisplatin has higher therapeutic activity and demonstrates a higher ORR, it does not show a change in survival advantage [4,8].

Therefore, from a clinical decision-making perspective, the choice between Cisplatin and Carboplatin needs to balance efficacy and toxicity. For example, in patients with NSCLC who have renal insufficiency, Carboplatin is often preferred due to its lower nephrotoxicity. In contrast, for patients with extensive-stage SCLC who need rapid symptom relief and are younger and healthier, the higher response rate of Cisplatin may be more advantageous [8,9]. However, the clinical application of Cisplatin and Carboplatin is greatly limited due to the cross-resistance between them.

#### 2.1.3. Second-Generation Derivative: Nedaplatin

**Figure 3:**
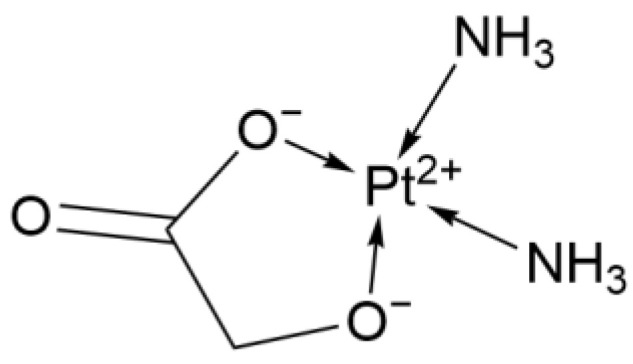
The structure of Nedaplatin.

Nedaplatin (NDP, Figure 3) was first approved in Japan in 1995 for the treatment of lung cancer and other solid tumors, and it has demonstrated significant clinical advantages. Its glycolate group replaces the chloride ligands of Cisplatin, giving Nedaplatin higher water solubility and a lower plasma-protein binding rate. These advantages are mainly reflected in lower non-hematologic toxicity and higher OS. However, it exhibits higher hematologic toxicity compared to Cisplatin.

A Phase III randomized controlled trial showed that in patients with advanced or recurrent squamous cell lung cancer, the median OS in the Nedaplatin combined with docetaxel group was significantly better than that in the Cisplatin group (13.6 months vs. 11.4 months). The incidence of non-hematologic toxicities such as Grade 3–4 nausea (4% vs. 14%), hyponatremia (14% vs. 30%), and hypokalemia (2% vs. 9%) was significantly reduced in the NDP group. However, NDP caused a higher incidence of neutropenia (82% vs. 70%) and thrombocytopenia (9% vs. 0%) [10]. Another retrospective study in NSCLC further confirmed that the median survival time (MST) in the NDP group (20 months vs. 15 months) and the 1-, 2-, and 3-year overall survival rates were all superior to those in the Cisplatin group. Moreover, patients in the Nedaplatin group had better tolerability, with significantly lower incidences of Grade 3–4 nausea/vomiting (8.4% vs. 36.1%), anorexia (5.8% vs. 17.3%), and weight loss (1.0% vs. 9.9%). However, Nedaplatin caused more severe thrombocytopenia (12.1% vs. 5.4%) [11]. In addition, another clinical trial for first-line treatment of NSCLC also showed that the response rate of NDP combined with chemotherapy (35.7%) was significantly higher than that of the Cisplatin group (17.1%), but it also exhibited a higher incidence of liver damage [12]. The above data indicate that NDP provides a more tolerable and clinically beneficial treatment option for lung cancer patients by optimizing the non-hematologic toxicity profile and extending OS. However, given its higher hematologic toxicity, clinicians still need to exercise caution when selecting medications for patients.

In addition, Nedaplatin reverses Cisplatin resistance by downregulating the expression of MVIH, thereby increasing the apoptosis rate of the Cisplatin-resistant cell line A549/DDP. This mechanism helps to overcome the drug resistance caused by traditional Cisplatin-based treatment regimens to a certain extent [13].

#### 2.1.4. Third Generation: Oxaliplatin

**Figure 4:**
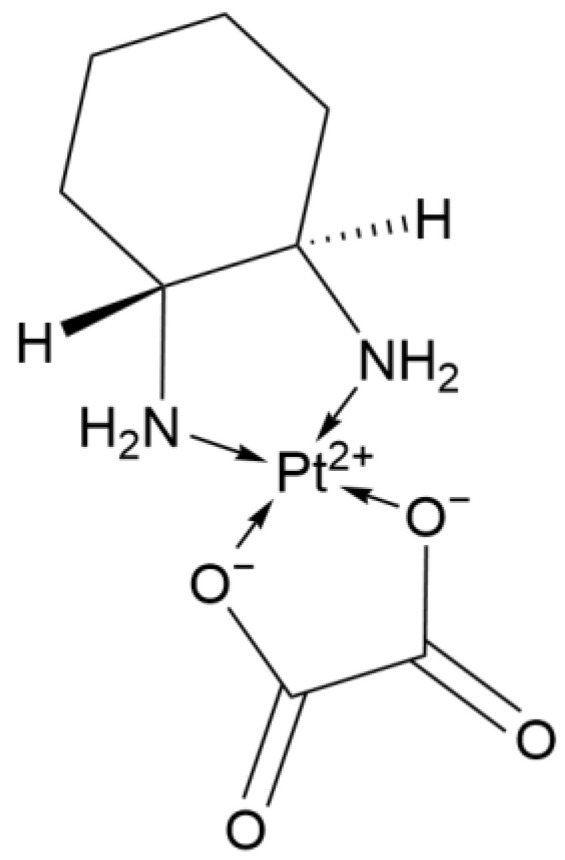
The structure of Oxaliplatin.

Platinum-based chemotherapeutic complexes often cause neurotoxicity. Despite the introduction of the cyclohexane ligand, the incidence of peripheral neuropathy (PN) caused by Oxaliplatin (Figure 4) is as high as 45%. However, unlike the irreversible damage caused by Cisplatin, the PN caused by Oxaliplatin is usually completely reversible within 6 to 8 weeks after discontinuation of the drug.

Studies have shown that when Oxaliplatin is used as a single agent for advanced NSCLC, the ORR is 15% [14]. When combined with vinorelbine for stage IV NSCLC, the ORR can be increased to 26% [15]. When combined with etoposide in elderly patients with extensive-stage SCLC, the ORR can reach 55.9%, which is comparable to the ORR of 54.3% when Cisplatin is combined with etoposide [16]. However, the toxicity of Oxaliplatin is significantly reduced: compared with the Cisplatin plus etoposide regimen, in which 8.3% of patients experienced renal impairment and 97.2% of patients experienced nausea/vomiting, no renal toxicity has been reported with Oxaliplatin, and the incidence of gastrointestinal reactions is significantly lower (65.7% in the Oxaliplatin plus etoposide group) [16].

Despite Oxaliplatin causing cold-related sensory abnormalities (in 69% to 88% of patients) and cumulative peripheral neuropathy (with a Grade 3 incidence of 15%), most of these symptoms are mild to moderate and reversible, and do not affect the continuity of treatment. It is worth noting that in patients with contraindications to Cisplatin (such as those with renal insufficiency, WHO performance status score ≥ 2, or elderly patients), Oxaliplatin-based combination regimens can still maintain dose intensity and achieve a median OS of 9.1 to 10.5 months [15].

These data indicate that Oxaliplatin significantly improves patient tolerance by avoiding the nephrotoxicity and severe gastrointestinal reactions associated with traditional platinum complexes, providing an equivalent and safer platinum treatment option for special populations. However, the management of its neurotoxicity still needs to be further explored through dose optimization or combination with neuroprotective strategies.

#### 2.1.5. Third-Generation Derivative: Lobaplatin

**Figure 5:**
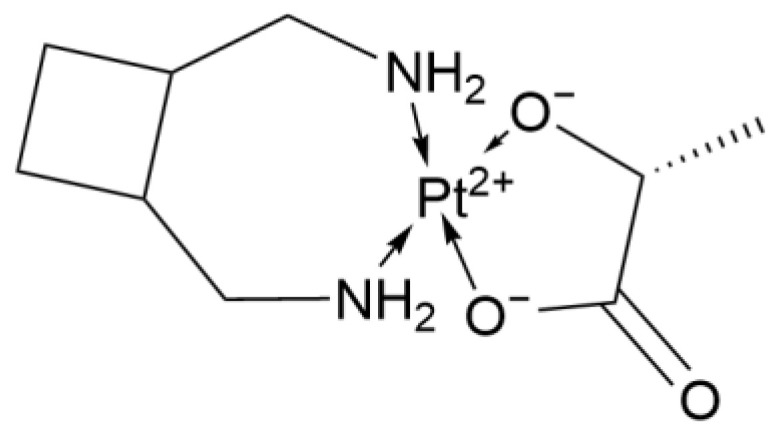
The structure of Lobaplatin.

Lobaplatin (LBP, Figure 5) is a third-generation platinum-based chemotherapeutic complex. In the treatment of lung cancer, it demonstrates antitumor activity comparable to that of previous generations of drugs (such as Cisplatin and Carboplatin). However, compared with earlier drugs, it significantly reduces non-hematologic toxicity, thereby providing more options for clinical treatment.

A randomized controlled trial showed that in patients with extensive-stage small cell lung cancer (ES-SCLC), the combination of Lobaplatin and etoposide, compared with the combination of Cisplatin and etoposide, had similar median progression-free survival (PFS) (5.1 months vs. 5.3 months) and OS (10.6 months vs. 9.7 months). However, the Lobaplatin regimen significantly reduced nephrotoxicity (2.5% vs. 11.7%) and gastrointestinal reactions (nausea 22.3% vs. 40.5%, vomiting 14.1% vs. 35.1%) [17]. In concurrent chemoradiotherapy for limited-stage small cell lung cancer (LS-SCLC), the Lobaplatin regimen had slightly lower 1-year, 2-year, and 3-year PFS compared with the Cisplatin regimen, but the 1-year, 2-year, and 3-year OS was similar to that of the Cisplatin regimen, and the incidence of radiation esophagitis was reduced. This indicates that Lobaplatin can effectively reduce non-hematologic toxicity [18].

However, the main dose-limiting toxicity of Lobaplatin is hematologic toxicity, which is usually manifested as neutropenia, leukopenia, anemia, and thrombocytopenia [19]. Nevertheless, a Phase II clinical trial indicated that hematologic toxicity can be effectively controlled through individualized dose adjustment based on creatinine clearance (Ccr). In addition, in elderly patients with SCLC, Lobaplatin achieved a high disease control rate of 80% and significantly prolonged the SR to 38.5% [20].

In summary, Lobaplatin has fewer adverse reactions and performs well in prolonging patients’ survival and controlling the disease(Table 2).

### 2.2. Platinum-Based Complexes in the Clinical Trial Stage

#### 2.2.1. Dicycloplatin

**Figure 6:**
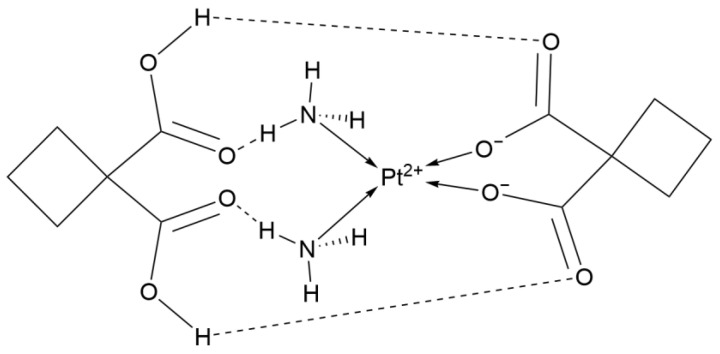
The structure of Dicycloplatin.

Dicycloplatin (DCP, Figure 6) is a third-generation platinum-based antitumor complex independently developed in China. Its unique hydrogen-bonded supramolecular structure and cyclobutane dicarboxylic acid structure provide it with significant advantages in terms of water solubility and stability [21].

Preclinical studies have shown that in the NSCLC cell line A549 xenograft model, the tumor growth inhibition rate of Dicycloplatin (67–90%) is significantly higher than that of Carboplatin (51–63%). Phase I clinical trials have shown that the dose-limiting toxicities of Dicycloplatin include thrombocytopenia, anemia, and vomiting [22]. Pharmacokinetics shows that the platinum plasma concentration of Dicycloplatin rapidly decreases within the first 4 h and then declines slowly over the long term. Its steady-state apparent volume of distribution is low, indicating stronger tissue targeting [23]. In a Phase II clinical trial, Dicycloplatin combined with paclitaxel was used to treat advanced NSCLC and was compared with Carboplatin combined with paclitaxel. The 1-year SR of the Dicycloplatin regimen reached 54.8%, which was higher than the 20.1% of the Carboplatin regimen. In another clinical trial with consistent treatment regimens and similar patient conditions, there were no statistical differences in several data, including the median overall survival and the 1-year SR between the two regimens. However, the 3-year SR of the Dicycloplatin regimen was 22.2%, which was also higher than that of the Carboplatin regimen (11.1%). Lymphocytopenia and myelosuppression were more common in the Carboplatin regimen, and other manifestations were basically consistent. This indicates that the effects of the two regimens are almost the same, with Dicycloplatin being slightly superior to Carboplatin [22].

These data indicate that Dicycloplatin, while maintaining the core antitumor mechanisms of platinum complexes, has achieved a breakthrough balance between efficacy and safety through molecular design and pharmacokinetic optimization, providing a new direction for the clinical iteration of platinum complexes [24].

#### 2.2.2. Satraplatin

**Figure 7:**
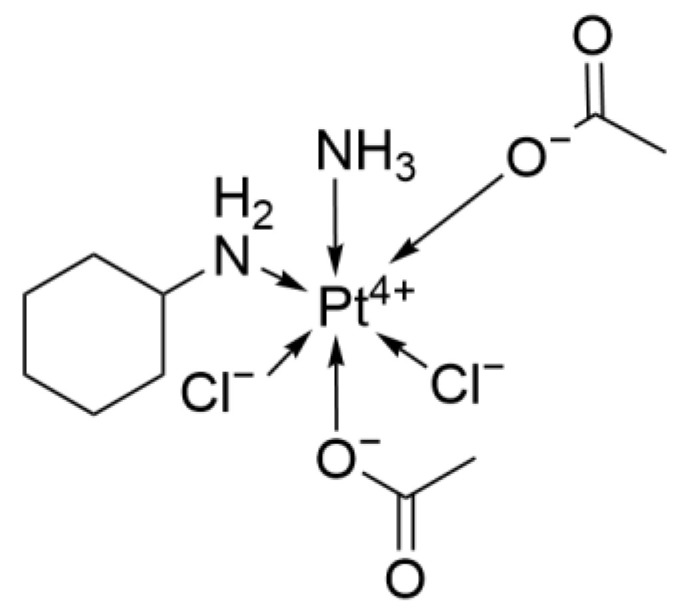
The structure of Satraplatin.

Satraplatin (Figure 7), systematically named cis-dichloro-trans-diacetato-ammine-cyclohexylamine-platinum(IV), is an oral formulation developed by Spectrum Pharmaceuticals (USA) and represents the fourth generation of platinum analogs. It is the first oral platinum candidate to follow the successful clinical application of Carboplatin, designed to circumvent the toxicity associated with intravenous infusion.

Notably, Satraplatin’s stable ligands alter the distribution of DNA adducts while retaining a similar mechanism of action, more effectively suppressing DNA mismatch repair and, to some extent, overcoming Cisplatin resistance.

The key innovation of Satraplatin lies in its chemical structure, which incorporates cyclohexylamine and acetate ligands. This enhances lipophilicity and stability, improves gastrointestinal absorption, enables oral administration, and reduces the vascular and tissue irritation caused by the high concentrations required for intravenous injection [25].

In terms of efficacy, Satraplatin and etoposide act synergistically. In a phase II trial involving patients with SCLC, the Satraplatin-containing regimen achieved an ORR of 38% (10 of 26 patients), comparable to that reported with single-agent Cisplatin [26]. In another phase II study of advanced NSCLC, Satraplatin failed to demonstrate any objective responses, performing worse than both Cisplatin (20%) and Carboplatin (12%) [25].

In summary, as the first oral platinum candidate, Satraplatin successfully avoids the toxicities associated with intravenous administration; however, its development is currently on hold.

#### 2.2.3. Lipoplatin

Lipoplatin was developed by the Greek company Regulon. It encapsulates Cisplatin inside liposomes, creating an innovative, targeted delivery system that reduces drug accumulation in healthy tissues while exploiting the EPR (enhanced permeability and retention) effect to achieve high concentrations in tumors.

Researchers encapsulated Cisplatin into ~110 nm liposome nanoparticles. Leveraging EPR-mediated targeting, these nanoparticles accumulate preferentially in tumors and metastases—surgical specimens showed Lipoplatin levels in tumor and metastatic tissue up to 200-fold higher than in adjacent normal tissue. Once at the tumor site, the liposomes either fuse directly with the cancer cell membrane or are taken up via endocytosis, bypassing the Ctr1 transporter normally required for Cisplatin entry. This circumvents one mechanism of Cisplatin resistance. Inside the cell, Cisplatin is released, forms DNA adducts, and exerts its cytotoxic effect [27].

Clinically, data from four NSCLC trials show that, compared with conventional Cisplatin, Lipoplatin significantly reduced the rate of progressive disease (PD) and increased the rate of partial responses (PR), while showing no clear advantage for stable disease (SD). This indicates that Lipoplatin achieves better tumor shrinkage while still maintaining disease stability [28]. In a phase II NSCLC trial, the Lipoplatin arm showed significantly lower incidences of nephrotoxicity, nausea/vomiting, and fatigue compared with the Cisplatin arm, confirming its core “reduced-toxicity” advantage [29].

#### 2.2.4. LA-12

**Figure 8:**
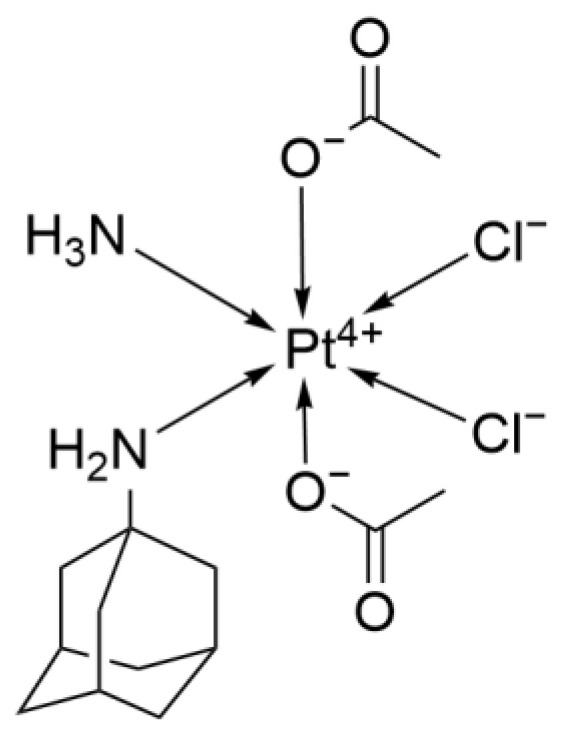
The structure of LA-12.

LA-12 (Figure 8) is a diacetyl-ammino-dichloro(adamantylamine) platinum(IV) complex. The ingenuity of its design lies in conjugating the adamantylamine moiety to the platinum center, enabling the two components to act synergistically against tumors and offering fresh inspiration for medicinal-chemistry innovation [30].

Thanks to its adamantane scaffold, LA-12 is markedly more lipophilic, which greatly facilitates cellular uptake. In an in vitro study, H1299 lung cancer cells accumulated 40–50 times more platinum from LA-12 than from an equimolar dose of Cisplatin, reaching peak levels rapidly and maintaining them steadily—demonstrating excellent penetration into lung cancer cells [31].

In p53-wild-type cell lines such as A549, low-dose LA-12 (1–5 μM) induces phosphorylation of p53 at Ser15 and causes a modest up-regulation of its downstream target p21WAF1/CIP1, whereas Cisplatin elicits markedly higher p21WAF1/CIP1 levels, indicating that Cisplatin more potently activates p53 transcriptional activity.

In p53-mutant or -deficient cells (e.g., the NSCLC line H1299), intracellular LA-12 binds with high affinity to the molecular chaperone Hsp90 (50 μM LA-12: 0.222 mg Pt/mg protein vs. <0.02 mg Pt/mg protein for Cisplatin). This interaction disrupts Hsp90–client-protein interactions and functionally suppresses mutant p53. Concomitantly, LA-12 triggers p53-independent apoptosis by up-regulating the pro-apoptotic genes Puma and Noxa, thereby directly activating the mitochondrial death pathway. These data indicate that LA-12 retains robust activity in Cisplatin-resistant tumor cells [31,32]. Moreover, Hsp90 inhibition promotes the degradation of the cell-cycle protein Cyclin D1, thereby blocking lung cancer cells from progressing from G1 to S phase and halting tumor cell division [31]. In addition, studies have shown that LA-12 up-regulates retinol-binding protein 4 (RBP4), thereby enhancing retinol transport and intracellular signaling. As a differentiation-inducing molecule, retinol can drive cancer cells toward a more normal phenotype and curb their limitless proliferative capacity. Although this mechanism has not yet been validated in lung cancer models, it may still offer a valuable direction for future investigations into LA-12’s therapeutic potential in lung cancer [33].

In vitro efficacy studies show that after 24 h continuous exposure, LA-12 displays markedly lower IC_50_ values than Cisplatin: 6 μM versus 63 μM for the lung cancer line A427, and 25 μM versus >80 μM for the Cisplatin-resistant large-cell carcinoma COR-L23/CTR. These data demonstrate LA-12’s potent cytotoxic activity against both parental and Cisplatin-resistant lung cancer cells, underscoring its potential as a future therapeutic option in lung cancer treatment [30].

Although most current data indicate that LA-12 remains in the preclinical stage, it holds significant potential to address the major clinical challenge of Cisplatin resistance.

#### 2.2.5. Pt-Exo

Researchers generated and isolated empty exosomes from MDA-MB-231 cells [34] and subsequently loaded them with Cisplatin and an anti-CD47 antibody. The goal is to achieve targeted killing of lung-tumor cells by Cisplatin while simultaneously activating an immune response that enables the immune system to eradicate the targeted cancer cells.

After entering the bloodstream, the exosomes rely on integrinβ4 on their surface to bind specifically to surfactant protein C (SPC) on A549 cells [35], achieving lung cancer-specific targeting. The exosomes are then internalized by the tumor cells, releasing their dual cargo—Cisplatin and an anti-CD47 antibody. Inside the cancer cells, Cisplatin forms DNA adducts. Concurrently, the anti-CD47 antibody competitively binds CD47 on the tumor cell membrane, disrupting the CD47–SIRPα axis and lifting the “don’t eat me” signal, thereby enabling macrophage-mediated phagocytosis of the tumor cells.

Following phagocytosis, macrophages secrete immune-stimulatory cytokines such as IL-12p70 and IFN-γ while down-regulating the immunosuppressive factor TGF-β. IL-12p70 and IFN-γ promote CD8^+^ T-cell proliferation, amplifying the antitumor immune response and eliminating residual tumor cells.

Moreover, the exosome-guided delivery markedly reduces Cisplatin accumulation in normal tissues (e.g., kidneys and gastrointestinal tract), thereby lowering systemic toxicities such as nephrotoxicity and nausea/vomiting associated with conventional chemotherapy. This strategy offers fresh inspiration for the overarching goal of “higher efficacy with lower toxicity.”

#### 2.2.6. Pt(II) Saccharin Complex

**Figure 9:**
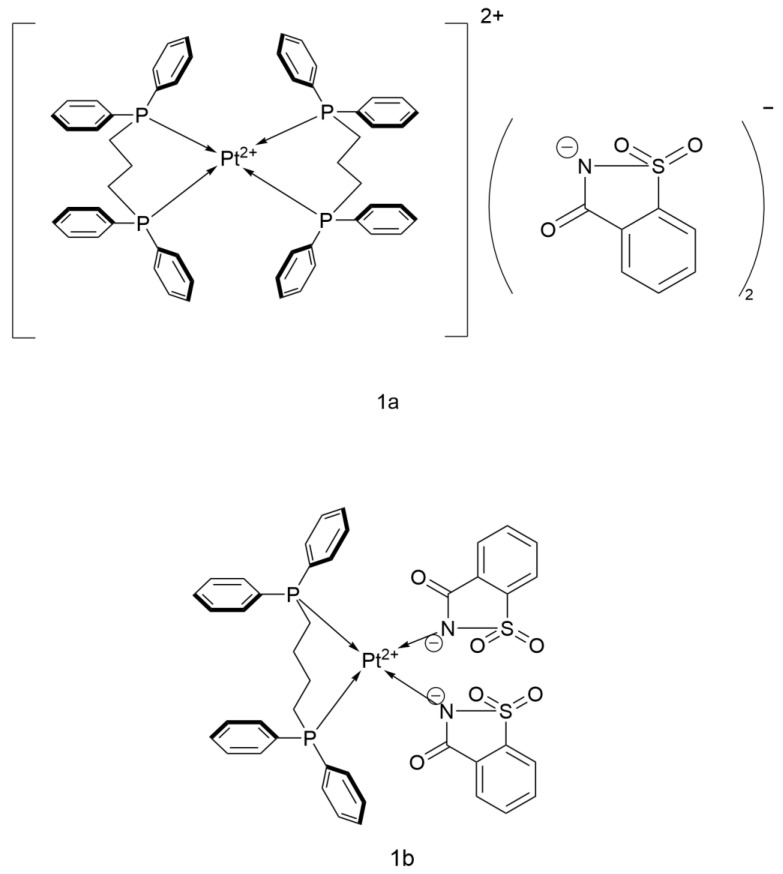
The structure of Pt(II) saccharin complex.

The Yilmaz research team designed a chelation ring (to enhance the interaction between the metal center and DNA) combined with saccharin anion coordination, which significantly improved the antitumor activity (Figure 9). In their study, the compounds [Pt(dppp)_2_](sac)_2_·2DMF (1a) and [Pt(sac)_2_(dppb)]·DMSO (1b) were used as representative drugs. These compounds demonstrated significantly better therapeutic effects than Cisplatin in the control experiments.

1a features a bidentate chelating ligand, 1,3-bis(diphenylphosphino)propane (dppp), and saccharin anion (sac^−^) as its structural core. Despite its low lipophilicity (log P = −0.02), it exhibits high cellular uptake (33.8 pmol/106 A549), likely penetrating the cell membrane through electrostatic interactions due to its cationic nature. This complex has an IC_50_ value of 0.49 μM for A549 cells, significantly better than that of Cisplatin (17.23 μM), showing potential to replace Cisplatin as the first-line drug for lung cancer treatment.

1b is constructed with 1,4-bis(diphenylphosphino)butane (dppb) and saccharin anion to form a seven-membered chelation ring. Its high lipophilicity (log P = +1.02) drives its passive transmembrane transport. It demonstrates strong antitumor activity, with an IC_50_ of 7.32 μM for A549 (2.4 times that of Cisplatin). Its mechanism of action is centered on apoptosis induction, triggering cell death through ROS overload, mitochondrial membrane depolarization, and DNA double-strand breaks (confirmed by γ-H2AX fluorescence labeling) [36].

#### 2.2.7. Fluorinated Bipyridine Cisplatin Analogs

**Figure 10:**
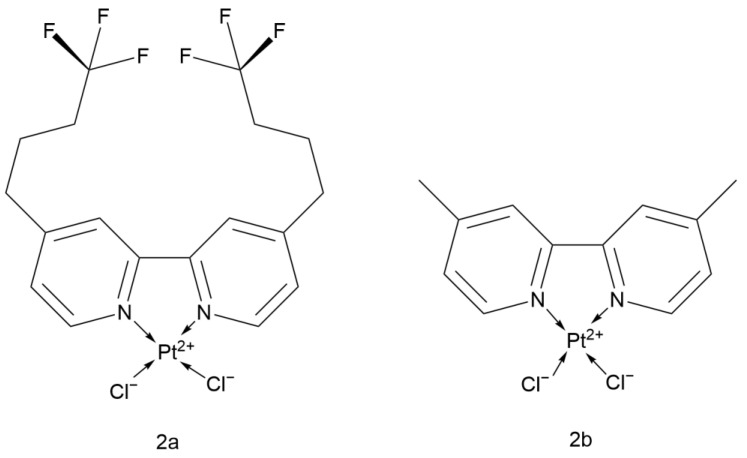
The structure of Fluorinated bipyridine Cisplatin analogs.

Elwell K. E. and colleagues synthesized two novel fluorinated bipyridine Cisplatin analogs (Figure 10): dichloro [4,4′-bis(4,4,4-trifluorobutyl)-2,2′-bipyridine]platinum (with trifluorinated alkyl substituents) and dichloro[4,4′-dimethyl-2,2′-bipyridine]platinum (with methyl substituents) (2a and 2b).

The cytotoxicity of 2a is significantly better than that of Cisplatin, with an EC_50_ value of 7.0 μM against A549 cells, which is 93% lower than that of Cisplatin (100.8 μM). The EC_50_ value of 2b was not specifically mentioned. Both compounds kill tumor cells by inducing apoptosis rather than necrosis. DNA damage caused by Cisplatin is usually repaired by the nucleotide excision repair (NER) pathway, which is also one of the mechanisms of Cisplatin resistance. 2a and 2b, with their 2,2′-bipyridine ligands, may block the binding of NER proteins, thereby improving the problem of resistance. Given its excellent performance, 2a can be further studied for its mechanism of action in tumor cells and its toxicity to normal cells to further assess its research prospects [37].

#### 2.2.8. Cisplatin Analog with 4,4′-Dialkoxy-2,2′-bipyridine

**Figure 11:**
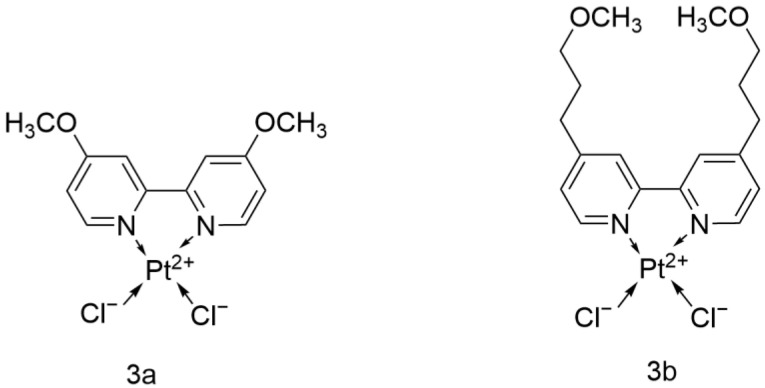
The structure of Cisplatin analog with 4,4′-dialkoxy-2,2′-bipyridine.

The Vo team systematically modified the ligand structure of Cisplatin and designed and evaluated two novel platinum-based anticancer complexes (Figure 11): dichloro(4,4′-bis[methoxy]-2,2′-bipyridine)platinum and dichloro(4,4′-bis[3-methoxy-n-propyl]-2,2′-bipyridine)platinum (referred to as 3a and 3b, respectively). In the study, a 1-h drug treatment was used, yielding an EC_50_ of 900 μM for Cisplatin.

Literature shows that among several tumor cell lines, A549 exhibits greater resistance to these two compounds. However, both 3a and 3b outperformed Cisplatin. Compared with Cisplatin, 3b showed better antitumor activity against A549, with an EC_50_ of 25 μM after only 1 h of incubation. Its structural long-chain ether group (-CH_2_CH_2_CH_2_OCH_3_) significantly enhanced its lipophilicity and demonstrated better radiosensitizing effects than Cisplatin, which can be combined with radiotherapy to further improve efficacy.

Both 3a and 3b possess the same bipyridine ligand as 2a and 2b and have shown excellent antitumor activity, which can further deepen the research on this ligand [38].

#### 2.2.9. BBR 3464

**Figure 12:**
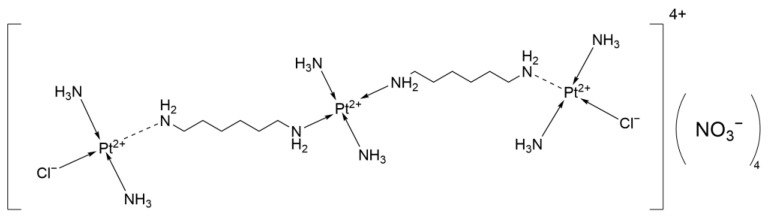
The structure of BBR 3464.

BBR 3464 (Figure 12) is a novel trinuclear platinum-based anticancer complex with the molecular formula C_12_H_40_Cl_2_N_11_O_3_Pt_3_^3+^. It has demonstrated antitumor activity in tumor models that are both sensitive and resistant to Cisplatin, as well as in models with p53 mutations. In Phase I trials, adverse reactions included myelosuppression and diarrhea. In a Phase I study to determine the maximum tolerated dose, out of 14 patients, 11 experienced severe neutropenia, which led to the early termination of the trial [39].

In a Phase II trial involving 37 patients with refractory small cell lung cancer (SCLC), Grade 3/4 hematologic toxicities were observed, including neutropenia (62%), febrile neutropenia (16%), anemia (10%), fatigue (5%), and hypokalemia (5%). No responses were observed in the 34 evaluable patients. The disease stabilization rate was 32% (35% in the sensitive group and 24% in the resistant group). The median time to progression (TTP) was 53 days (resistant) and 66 days (sensitive), while the median OS was 78 days (resistant) and 209 days (sensitive). It can be seen that it only showed effects in stabilizing the disease process.

BBR 3464 lacks activity as a single agent in recurrent SCLC and has significant toxicity, and does not support further development as a monotherapy [40].

#### 2.2.10. Picoplatin

**Figure 13:**
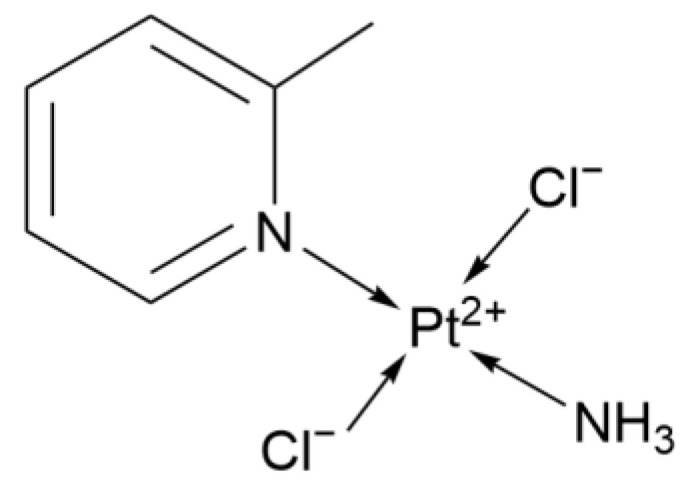
The structure of Picoplatin.

Picoplatin (Figure 13) is a monofunctional cationic platinum(II) complex named cis-diammine(pyridine)chloroplatinum(II). It exerts its antitumor activity through monofunctional DNA binding and uptake pathways dependent on organic cation transporters (OCTs), thus having a different mechanism of action from traditional bifunctional platinum complexes (such as Cisplatin and Oxaliplatin).

Preclinical studies have shown that Picoplatin exhibits time-dependent anticancer activity in NSCLC cell lines. In HOP-62 cells, the IC_50_ gradually decreases from 1 to 72 h, reaching the lowest level (24 μmol/L) after 72 h. High expression of ERCC1 is associated with Cisplatin resistance, while the level of ERCC1 decreases after treatment with Picoplatin. Moreover, Picoplatin-DNA adducts can evade the nucleotide excision repair pathway, holding promise for overcoming Cisplatin resistance [41]. In Phase II clinical trials, Picoplatin showed only a 4% partial response (PR) in SCLC patients who were resistant to platinum complexes. The median PFS and OS were 9.1 weeks and 26.9 weeks, respectively, which were significantly lower than the efficacy of traditional platinum complexes in second-line treatment [42]. However, pharmacokinetic limitations (low oral bioavailability and insufficient plasma concentration) and the failure to address the core issue of platinum resistance have restricted its clinical value [43].

In summary, although Picoplatin is innovative in its mechanism of action, its clinical activity and survival benefit have not met expectations. This suggests that further optimization of drug design or exploration of combination therapy strategies is needed to break through the efficacy limitations of current platinum-based complexes.

#### 2.2.11. Aerosolized SLIT Cisplatin

Sustained Release Lipid Inhalation Targeting Cisplatin (SLIT Cisplatin) is an inhalable Cisplatin formulation based on liposomal encapsulation technology. It delivers the drug to pulmonary lesions in a targeted and sustained-release manner through nebulization, thereby prolonging the retention time of Cisplatin in lung tissue.

At the maximum dose, SLIT Cisplatin did not exhibit dose-limiting toxicity (DLT). Only with prolonged repeated inhalation would the platinum concentration in the plasma slightly increase, thus avoiding some of the toxicities commonly associated with traditional chemotherapy, such as hematologic toxicity, nephrotoxicity, and ototoxicity. The main adverse reactions were reversible respiratory tract irritation symptoms, including dyspnea (64.7%) and hoarseness (47.1%).

Although the anticancer activity of Cisplatin after liposomal encapsulation was not weakened, the therapeutic effect was not satisfactory due to the difficulty in depositing the drug at the target site [44].

#### 2.2.12. Platinum(II) Cyclometalated Complexes Containing 2-Vinylpyridine

**Figure 14:**
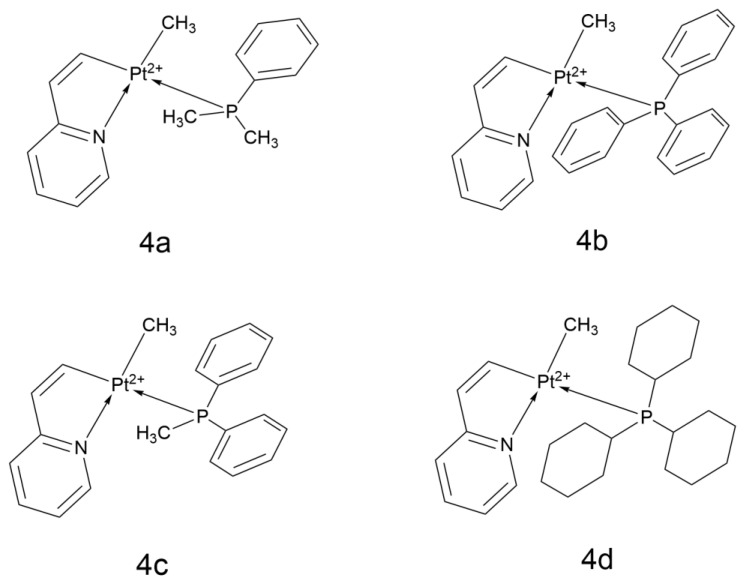
The structure of Platinum(II) cyclometalated complexes containing 2-vinylpyridine.

The study by Mojaddami et al. systematically evaluated the antiproliferative activity of four platinum(II) cyclometalated complexes containing 2-vinylpyridine ([PtMe(vpy)(L)], L = PMe_2_Ph (4a), PPh_3_(4b), PPh_2_Me (4c), PCy_3_(4d)) against A549 (Figure 14).

The four platinum(II) cyclometalated complexes containing 2-vinylpyridine and different phosphine ligands exhibited a wide range of antiproliferative activities against A549. 4a showed moderate cytotoxicity (IC_50_ = 24.94 μM, compared to 10.12 μM for Cisplatin) and a selectivity index (SI) of 1.70 (compared to 2.84 for Cisplatin), which may be related to hydrophobic interactions with minor groove bases of DNA (G4/G10). Although 4b exhibited strong binding affinity to B-DNA (ΔG = −9.91 kcal/mol), which can stabilize Pt-DNA adducts, its activity was relatively weak (IC_50_ = 49.48 μM) and further optimization is needed. 4c had the highest cytotoxicity (IC_50_ = 21.10 μM) and a multi-mechanistic synergistic effect, including minor groove binding (ΔG = −10.06 kcal/mol), induction of apoptosis, and ROS generation. Finally, 4d, containing the bulky tricyclohexylphosphine ligand, had low activity due to limited cellular uptake (IC_50_ = 91.89 μM) but retained the ability to form adducts with DNA (ΔG = −8.21 kcal/mol), suggesting potential for structural optimization [45].

#### 2.2.13. Quinolinecarboxaldehyde Selenosemicarbazone Pt(II) Complex

**Figure 15:**
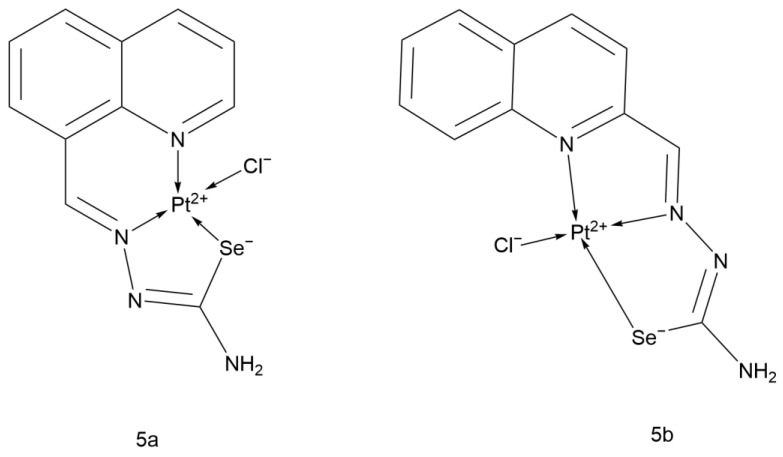
The structure of Quinolinecarboxaldehyde selenosemicarbazone Pt(II) complex.

Nenad Filipović and colleagues synthesized platinum(II) complexes of 8-quinoline-2-carboxaldehyde selenocyanate and 2-quinoline-2-carboxaldehyde selenocyanate (5a and 5b, Figure 15) and studied their anticancer activity against H460 cells. The ligands coordinate to platinum in a tridentate manner through the nitrogen atoms of the quinoline and azomethine groups and the selenium atom.

After 24 h of treatment, the IC_50_ of 5a against H460 cells was 46.3 μM, while 5b did not show significant anticancer activity. This indicates that the position of the quinoline-2-carboxaldehyde selenocyanate ligand plays a decisive role. Flow cytometry analysis of the cell cycle showed that after treatment with 5a, H460 cells accumulated in the M phase, demonstrating a different mechanism of action from Cisplatin.

In the radical scavenging assay, the ligand of 5a exhibited the highest capacity for scavenging cation radicals, but 5a did not inherit this activity. The research on the two compounds in the literature is not in-depth enough, and based on the existing data, 5a and 5b did not show outstanding performance, so there is little necessity for further in-depth research on them [46].

#### 2.2.14. Cisplatin-Derived Complexes of Selenones

**Figure 16:**
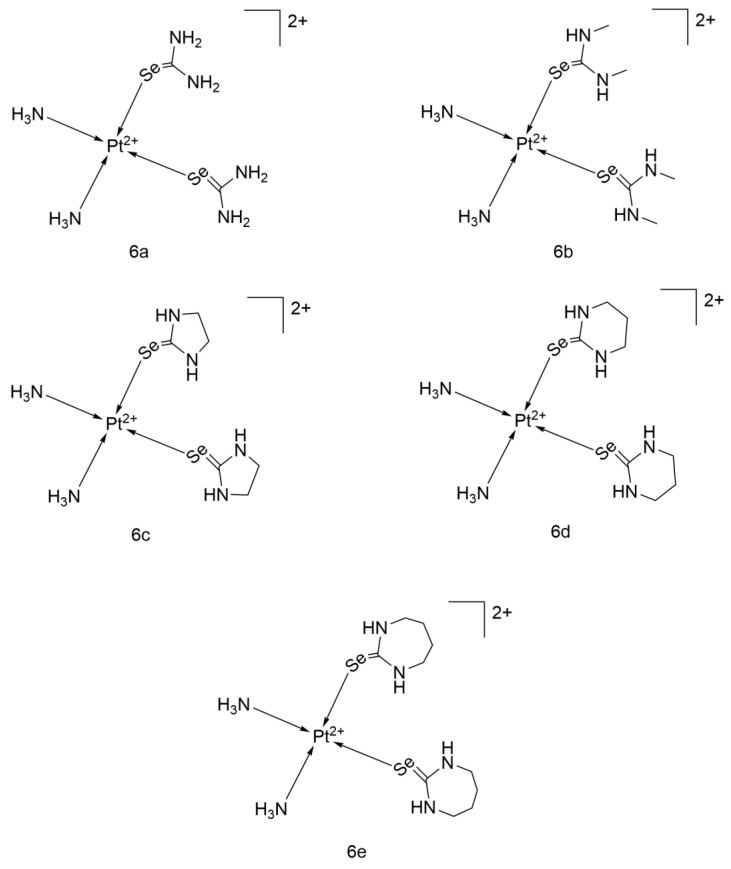
The structure of Cisplatin-derived complexes of selenones.

The Sobeai team designed and studied five different selenone ligand derivatives of Cisplatin: [Pt(NH_3_)_2_(Seu)_2_](NO_3_)_2_, [Pt(NH_3_)_2_(Me_2_Seu)_2_](NO_3_)_2_, [Pt(NH_3_)_2_(ImSe)_2_](NO_3_)_2_, [Pt(NH_3_)_2_(DiazSe)_2_](NO_3_)_2_, and [Pt(NH_3_)_2_(DiapSe)_2_](NO_3_)_2_ (6a–6e, Figure 16). All five compounds had negative binding energies with DNA, and 9e had the most negative value (−9.67 kcal/mol), indicating the highest affinity.

Cytotoxicity analysis against A549 showed that only 6c and 6d outperformed Cisplatin (IC_50_ = 1.20 μM vs. 6.74 μM), while 6a and 6b were virtually inactive, and 6e had some activity (IC_50_ = 54.02 μM). Further research on the best-performing 6c revealed that in A549 treated with 1.2 μM of 6c, the expression of 32 miRNAs was affected. The activity of PI3K, which is associated with tumor resistance and poor prognosis, decreased, with the proportion of PI3K-active cells dropping from 39.91% to 27.6%. This may be the molecular mechanism underlying its efficient tumor cell killing. 6c significantly induced apoptosis in a dose-dependent manner, with an apoptosis rate of 87.8% after 24 h of treatment at a dose of 30 μM.

Although 6d and 6e both had better DNA binding energies and related inhibition constants (Ki) than 6c, their IC_50_ values were still lower than that of 6c. This may be due to their larger molecular volumes, which increase spatial hindrance and reduce cellular uptake efficiency.

Overall, selenone ligands performed better than Seu. 9c has great potential to become a first-line drug for lung cancer treatment and also provides valuable research and development experience for subsequent efforts to inhibit PI3K activity and regulate cancer cell miRNA expression [47].

#### 2.2.15. Mon-Pt-2

**Figure 17:**
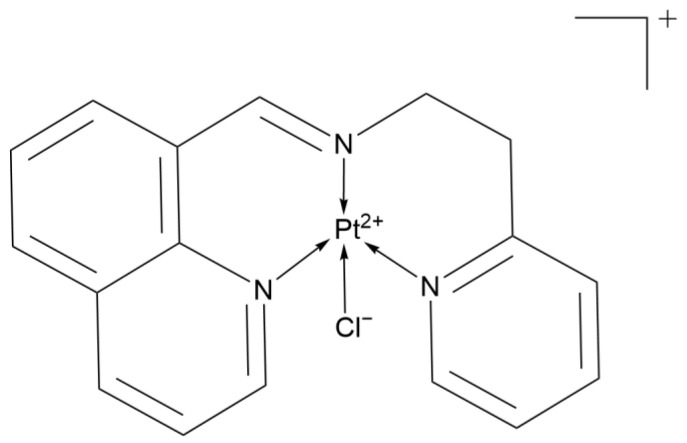
The structure of Mon-Pt-2.

The Feng-Yang team designed Mon-Pt-2 (Figure 17) with 8-substituted quinoline derivatives as ligands, aiming to coordinate the dual pathways of apoptosis and autophagy induction to jointly cause lung cancer cell death.

Literature indicates that Mon-Pt-2 continuously accumulates in the mitochondria of A549 cells, leading to mitochondrial membrane potential depolarization and decreased ATP levels. This triggers downstream reactive oxygen species (ROS) bursts and endoplasmic reticulum (ER) stress, acting as the “fuse” for cell death. The released ROS attack mitochondria, proteins, and DNA, exacerbating dysfunction. Moreover, the leaked ROS gather in the ER region, causing ER stress and upregulating the expression of ER stress markers PERK, elF2a, and CHOP.

Mon-Pt-2 has dual regulatory effects on promoting apoptosis and autophagy, forming a “double insurance” death mechanism: it inhibits Bcl-2 and upregulates Bax, promoting the release of mitochondrial cytochrome C, thereby activating the activity of caspase-9/3 and causing cell apoptosis. Mon-Pt-2 treatment stimulates the expression of total LC3 protein, and over time, the conversion of LC3-I to the autophagosome marker LC3-II significantly increases, confirming its autophagy-inducing effect.

These research findings suggest that Mon-Pt-2 induces cell death through dual pathways of apoptosis and autophagy via mitochondrial-ER interaction. Because it does not rely on the DNA binding pathway, it efficiently overcomes Cisplatin resistance and reduces off-target effects, holding promise as a potential first-line treatment drug in the future [48].

#### 2.2.16. 1,6-Naphthyridine Ligand

**Figure 18:**
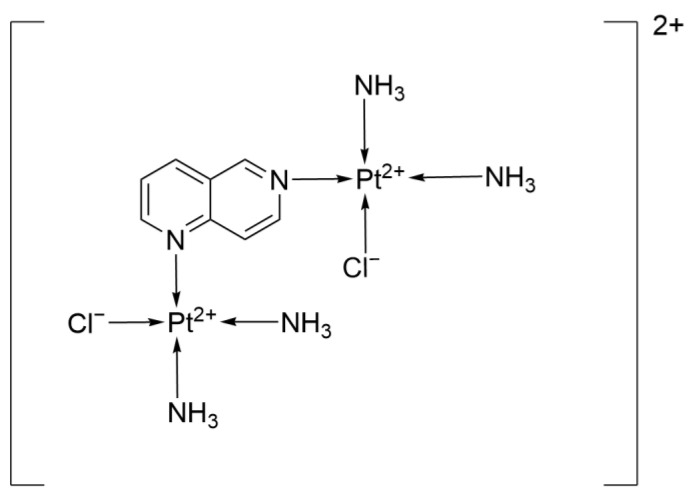
The structure of 1,6-naphthyridine ligand.

The dinuclear platinum(II) complex Pt-1 ({PtCl(NH_3_)_2_}_2_(μ-1,6-nphe)](ClO_4_)_2_) (Figure 18), which was synthesized and studied, features 1,6-naphthyridine as the bridging ligand. The two [PtCl(NH_3_)_2_] units form a stable dinuclear configuration through the synergistic action of the nitrogen-containing ring, exhibiting unique antitumor activity and characteristics of interaction with biomolecules.

Mechanistic studies have revealed that Pt-1 exerts its antitumor effects through dual pathways: on the one hand, it upregulates the pro-apoptotic protein caspase-3 (15.24% vs. 7.3%) and downregulates the anti-apoptotic protein Bcl-2 (10.9% vs. 19.7%), thereby inducing apoptosis from both aspects; on the other hand, it downregulates the expression of the cell proliferation marker Ki-67 (2.25% vs. 4.95%), thus inhibiting the proliferation of tumor cells. Moreover, according to the experimental results, Pt1 binds to DNA through electrostatic interactions, and its mechanism of binding with plasma albumin indicates that it can cross-link and damage DNA while extending the drug’s half-life in the blood and reducing its general toxicity through the albumin carrier.

In terms of therapeutic efficacy, although the IC_50_ of Pt-1 is 49.625 μM, which is much higher than that of Cisplatin (IC_50_ = 3.70 μM), its higher selectivity for normal cells mMSC (SI = 1.67 vs. Cisplatin’s 1.24) suggests that it still has the potential to become a first-line clinical drug [49].

### 2.3. Platinum-Based Complexes in the Laboratory Research Phase

#### 2.3.1. Platinum(II) Cyclopentadienyl-Dithiocarbamate Complexes

**Figure 19:**
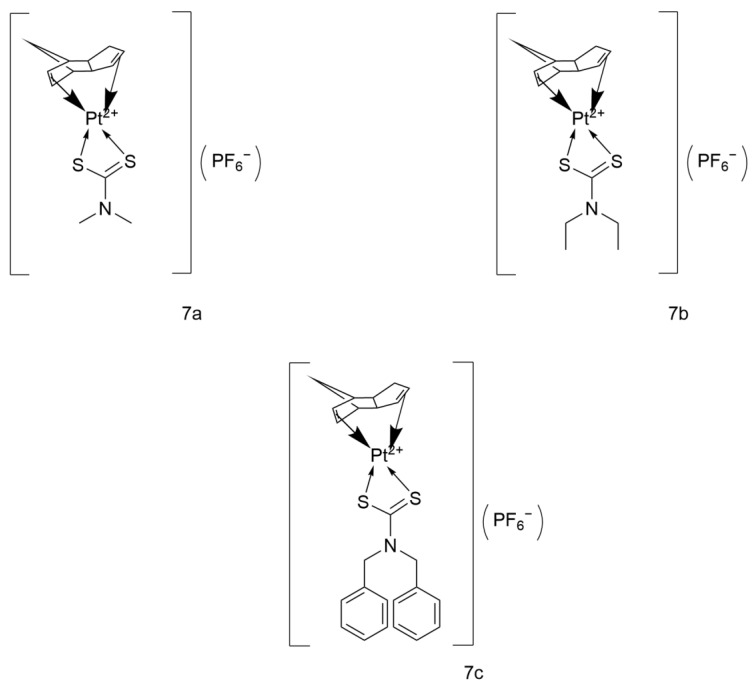
The structure of Platinum(II) cyclopentadienyl-dithiocarbamate complexes.

The three classes of platinum(II) cyclopentadienyl-dithiocarbamate complexes (Figure 19) designed by the Sulaiman team (namely [Pt(η^4^-DCP)(Me_2_DTC)]PF_6_, [Pt(η^4^-DCP)(Et_2_DTC)]PF_6_, and [Pt(η^4^-DCP)(Bz_2_DTC)]PF_6_, abbreviated as 7a–7c) demonstrate differentiated anti-lung cancer potential through a non-classical mechanism.

7a exhibits the highest activity in A549, with a 24-h IC_50_ of 7.61 μM (Cisplatin: 8.41 μM). Its activity is further enhanced at 72 h, with an IC_50_ of 3.37 μM (Cisplatin: 4.42 μM), representing a 24% increase in inhibition efficiency. Mechanistic studies reveal that it achieves multitarget synergistic effects through the mitochondrial apoptosis pathway (downregulation of BCL-2 and activation of caspase-3/7), G2/M cell cycle arrest, and induction of oxidative stress.

7c has an IC_50_ of 10.12 μM against A549, with activity comparable to Cisplatin. However, the high lipophilicity of benzyl dithiocarbamate (Bz_2_DTC) allows it to more easily penetrate tumor cell membranes or accumulate in lipid-rich tumor tissues. This results in higher local concentrations and prolonged drug exposure time, thereby delaying the metastasis of lung cancer and providing a potential therapeutic strategy for patients with advanced metastatic disease.

7b shows relatively weak direct inhibitory activity against A549, with an IC_50_ of 60.38 μM. However, the moderate steric hindrance of Et_2_DTC may balance lipophilicity and nephrotoxicity risk, making it suitable for patients with renal insufficiency.

These complexes break through the limitations of traditional platinum complexes through non-classical mechanisms. Their differentiated characteristics (high efficacy, potential for reversing resistance, and low toxicity) offer new ideas for the development of precision treatment strategies for lung cancer [50].

#### 2.3.2. Heterometallic Compounds

**Figure 20:**
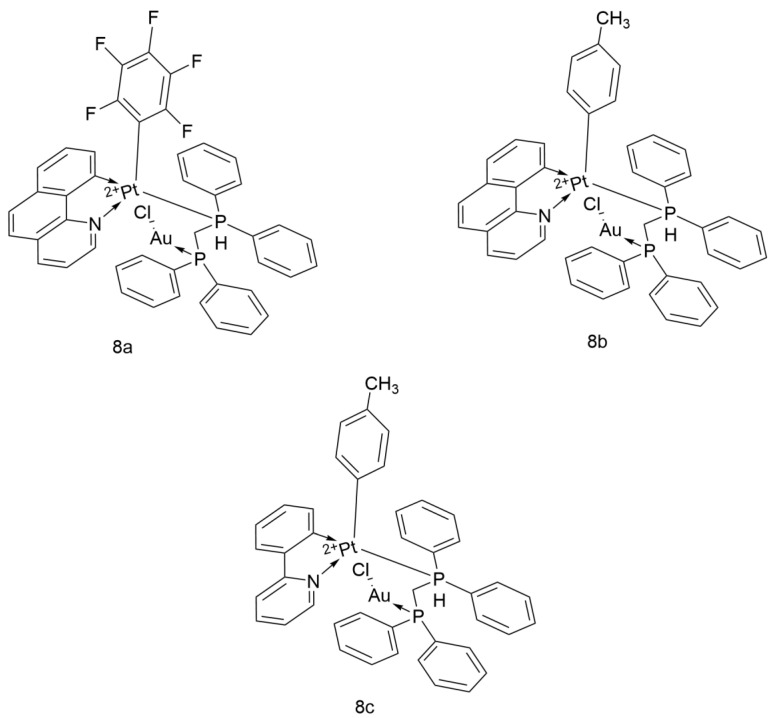
The structure of Heterometallic compounds.

The heterometallic Pt^2+^-Au^+^ complexes (Figure 20) developed by the Shahsavari team (namely [Pt(bzq)(C_6_F_5_)(μ-dppm)AuCl], [Pt(bzq)(p-MeC_6_H_4_)(μ-dppm)AuCl], and [Pt(ppy)(p-MeC_6_H_4_)(μ-dppm)AuCl], hereinafter referred to as compounds 8a, 8b, and 8c) have demonstrated efficient, selective, and multi-mechanistic anticancer properties against lung cancer tumors. Their unique application effects make them candidate molecules for novel anticancer drugs.

8a, with pentafluorophenyl (C_6_F_5_) and benzoxazole (bzq) as auxiliary ligands, has an IC_50_ of 86.32 μM against A549 cells, showing weaker antitumor activity compared to Cisplatin (9.63 μM). However, its weak luminescence in solution makes it possible to achieve localization in tumor cells. Further optimization of ligand design is needed to enhance its biological activity.

8b exhibits significant activity against A549 (IC_50_ = 5.47 μM), with an efficacy 1.8 times that of Cisplatin. Structural analysis indicates that the lipophilicity of the p-methylphenyl (p-MeC_6_H_4_) group facilitates cellular uptake of the drug. The extended π-conjugated structure of the benzoxazole (bzq) ligand stabilizes the mixed excited state dominated by ligand internal charge transfer (^3^IL) and metal-to-ligand charge transfer (^3^MLCT) through enhanced electron delocalization. This stable excited state significantly enhances anticancer activity by optimizing π-π stacking and charge complementarity between the molecule and its target (such as DNA). Additionally, it shows no significant toxicity to normal breast epithelial cells (MCF-10A, IC_50_ > 100 μM). These data suggest that it has the advantage of high activity and selectivity.

8c, with 2-phenylpyridine (ppy) as the cyclometalating ligand, has an IC_50_ of 8.51 μM against A549, which is slightly higher than that of compound 8b but still better than Cisplatin (IC_50_ = 9.63 μM). Notably, its toxicity to MCF-10A is low (IC_50_ > 100 μM).

The 8a–8c prodrugs lacking Au exhibit weaker cytotoxicity against tumor cells, while the IC_50_ is reduced after the addition of Au, indicating a synergistic effect between Pt and Au.

In summary, 8b stands out due to its high activity and selectivity. 8c has moderate activity but high selectivity, while compound 8a’s emission characteristics in solution allow for the assessment of its intracellular localization through fluorescence microscopy imaging. The multi-mechanistic actions (DNA damage, TrxR inhibition, and apoptosis induction) and luminescent properties of these complexes provide innovative strategies for targeted lung cancer therapy and real-time monitoring [51].

#### 2.3.3. Cyclometalated Platinum(II) Complexes with O,S-Heterocyclic Ligands

**Figure 21:**
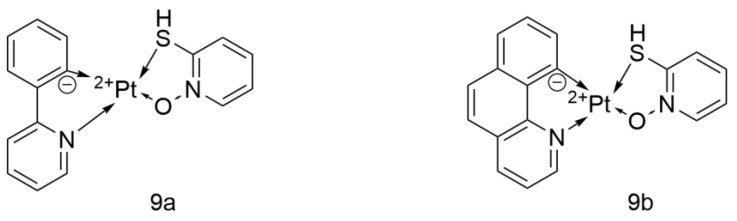
The structure of Cyclometalated Platinum(II) Complexes with O,S-heterocyclic ligands.

The Fredendal team designed two platinum(II) cyclometalated complexes (Figure 21), [Pt(ppy)(SpyO)] and [Pt(bzq)(SpyO)], which are hereafter referred to as 9a and 9b, respectively. Both complexes exhibited weak inhibitory activity against A549 (IC_50_ = 86.3 µM/59.1 µM), which was significantly inferior to that of Cisplatin (IC_50_ = 5.7 µM). In the study, the IC_50_ values of both complexes against MCF-12A were greater than 200 µM. However, Cisplatin also showed the same result, so the conclusion that “both complexes have high selectivity” needs to be reconsidered. In the future, their efficacy could be enhanced by targeted delivery, optimizing the structure to increase their accumulation in lung cancer tissues, or combining them with immunotherapy [52].

### 2.4. Innovations in Drug Delivery Systems of Platinum-Based Complexes

#### 2.4.1. AP 5280

**Figure 22:**
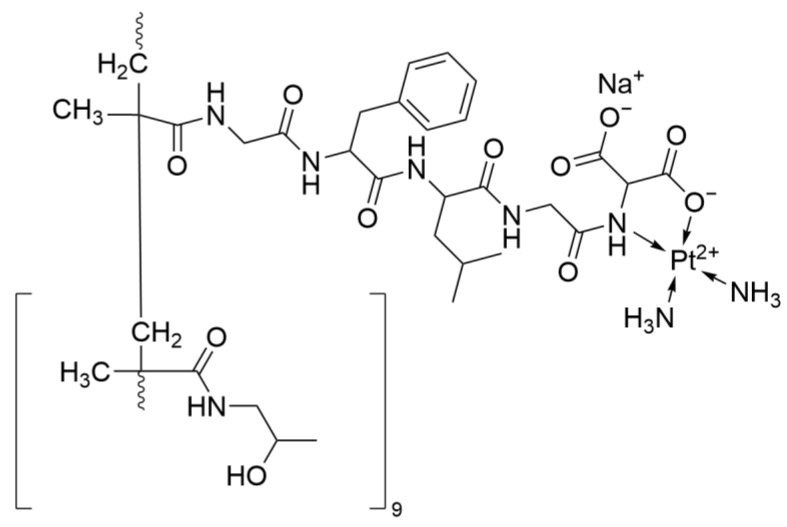
The structure of AP 5280.

AP 5280 (Figure 22) is a platinum complex conjugated with N-(2-hydroxypropyl)methacrylamide copolymer, utilizing the Enhanced Permeability and Retention (EPR) effect to achieve tumor-targeted delivery. This approach aims to increase the accumulation of platinum in tumor tissues while reducing systemic toxicity. Compared with traditional platinum complexes, the Maximum Tolerated Dose (MTD) of AP5280 is as high as 4500 mg Pt/m^2^, significantly higher than that of Cisplatin (100 mg/m^2^) and Carboplatin (360 mg/m^2^). Moreover, it has minimal hematologic toxicity.

Pharmacokinetic analysis shows that the total platinum plasma half-life of AP5280 is as long as 116 h, with only 4% of the total platinum being free platinum. In contrast, traditional Cisplatin has a higher proportion of free platinum and a half-life of only 1–2 h. The sustained-release characteristic of AP5280 may reduce systemic exposure.

In Phase I studies, monotherapy with AP5280 only achieved disease stabilization in 17% of patients, which may be related to insufficient effective concentration [53].

#### 2.4.2. SPI-77

SPI-77 is Cisplatin encapsulated in spatially stabilized liposomes, designed to reduce systemic exposure to free platinum. Theoretically, this method should enhance targeting to tumors and mitigate the classic toxicities associated with Cisplatin. However, like SLIT Cisplatin, clinical trials have shown that the efficacy of SPI-77 is not ideal.

Phase I studies indicated that the pharmacokinetics of SPI-77 are completely different from those of Cisplatin, with its metabolism in the body being dominated by the liposomes [54]. In a Phase II trial targeting advanced NSCLC, SPI-77 (at doses of 100–260 mg/m^2^) only demonstrated an ORR of 4.5% (1/22). The concentration of free platinum accounted for only 0.139% of the total platinum concentration in plasma. In terms of adverse reactions, the most significant toxicity was Grade 2 anemia in 31% of patients, indicating good tolerability. Only one patient experienced a 20-decibel unilateral hearing loss, whereas a study on the ototoxicity of traditional Cisplatin (at doses of 50–100 mg/m^2^) found that hearing loss was more frequent after treatment [55].

In a Phase II study involving NSCLC patients who were resistant to Cisplatin, SPI-77 (at a dose of 260 mg/m^2^) showed an ORR of 0% (0/12) and a disease stabilization rate (SD) of only 17% (2/12), with a median OS of 24.3 weeks. Pharmacokinetic analysis further revealed that the concentration of free platinum accounted for only 0.139% of the total plasma platinum. Given that the patients had previously received platinum-based chemotherapy, it is evident that SPI-77 performed worse in resistant patients. This suggests that the excessive stability of the liposomes may have hindered the timely release of free platinum, thereby limiting the drug’s bioavailability in the tumor microenvironment. This, in turn, led to reduced drug activity and toxicity, consistent with findings from previous literature [56].

Although SPI-77 has shown excellent performance in toxicity control compared with traditional Cisplatin, its efficacy is not sufficient to support its use as a first-line treatment for NSCLC.

#### 2.4.3. Platinum Nanoparticles

The Ismail team induced oxidative stress in A549 with 350 μM hydrogen peroxide (H_2_O_2_) to explore the antioxidant mechanism of 3-nm platinum nanoparticles (PtNPs). The experimental results showed that higher concentrations of PtNPs (100 μg/ml) significantly reduced the production of reactive oxygen species (ROS) and DNA damage. In addition, PtNPs enhanced the activity of superoxide dismutase (SOD), glutathione peroxidase (GPx), and catalase (CAT), thereby reversing the oxidative stress state. Transmission electron microscopy showed that PtNPs could penetrate the cell membrane and enter the nucleus within 3 h. After 24 h, they further accumulated in the cytoplasm and nucleus, demonstrating efficient cellular uptake and nuclear targeting ability. Although the antioxidant capacity of PtNPs was lower than that of ascorbic acid (determined by the FRAP assay), they exhibited better cellular activity during acute exposure (3 h) and could be used for neoadjuvant therapy or acute oxidative stress intervention [57].

In addition, PtNPs can also interact with cellular signaling pathways such as PI3K/AKT and MAPK, making cancer cells more sensitive to chemotherapy [58].

PtNPs may offer a less toxic adjuvant strategy for lung cancer treatment. However, their limitations include concentration-dependent effects, exposure time, and the stability of the nanoparticles. Currently, PtNPs have been used to enhance the radiation effects in tumor therapy.

#### 2.4.4. EG-Se/Pt

**Figure 23:**
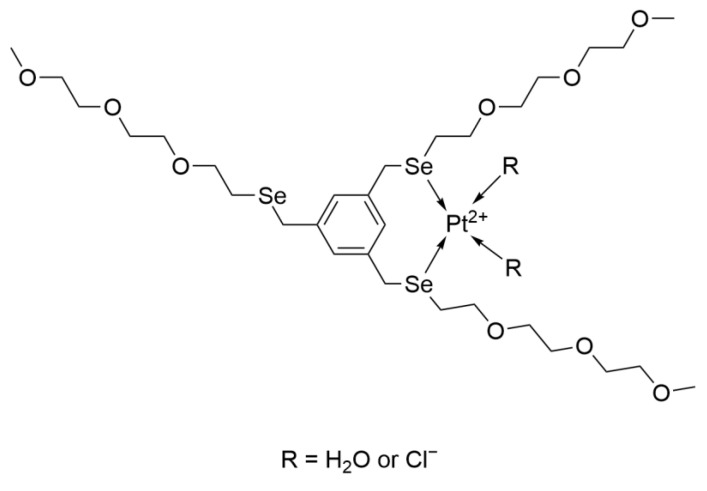
The structure of EG-Se/Pt.

The Zeng L team coordinated the selenium-containing small molecule EG-Se with Cisplatin to synthesize an amphiphilic platinum complex containing selenium, which was encapsulated by polyethylene glycol (Figure 23). This complex targets the glutathione antioxidant defense system to induce apoptosis in tumor cells.

Transmission electron microscopy images showed that EG-Se/Pt could self-assemble into spherical nanoparticles with sizes ranging from 100 to 500 nm. These nanoparticles have stealth properties that enable them to evade phagocytosis by macrophages, which is advantageous for drug action. In vivo experiments in rats demonstrated that approximately 20% of EG-Se/Pt remained in the bloodstream 24 h after administration. This experiment confirmed the aforementioned characteristics and verified the stability of the nanoparticle drug.

Moreover, compared with Cisplatin, EG-Se/Pt can interact with both the reduced (GSH) and oxidized (GSSG) forms of glutathione to inactivate them. This targets the glutathione antioxidant defense system, leading to excessive accumulation of ROS, depolarization of the mitochondrial membrane, and release of cytochrome C, which induces the mitochondrial apoptotic pathway. This redox homeostasis imbalance is selective, allowing better killing of tumor cells and improved safety. Additionally, in cytotoxicity analysis, the drug showed cytotoxic effects comparable to those of Cisplatin [59,60].

#### 2.4.5. Platinum Coordinated Elenomethionine

**Figure 24:**
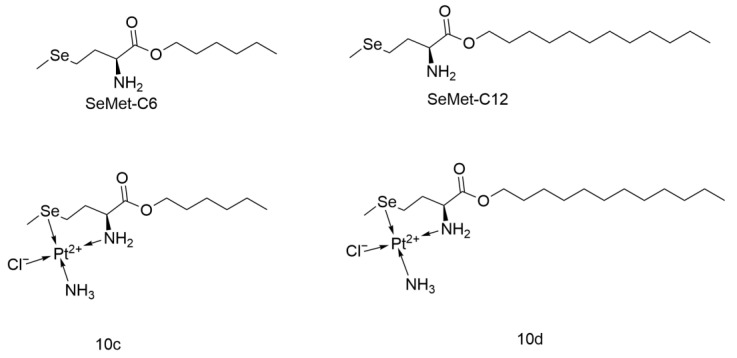
The structure of Platinum coordinated selenomethionine (present 10c and 10d).

Tianyu Li and colleagues synthesized a series of platinum-coordinated selenomethionine complexes (SeMet-TEG/Pt, SeMet-C2/Pt, SeMet-C6/Pt, SeMet-C12/Pt, referred to as 10a-10d) using selenomethionine esters and Cisplatin, which have self-assembly properties that can modulate their anticancer activity (Figure 24).

From the perspective of self-assembly, 10a and 10b could not form regular aggregates at low concentrations, while the aggregates formed by 10c and 10d were smaller than the original ligand size. 10d, composed of a hydrophilic selenium-platinum coordination part and a hydrophobic dodecyl part, could form spherical aggregates similar to its ligand, thus 10c and 10d showed higher potential.

In the in vitro cytotoxicity experiments on A549 cells, 10c and 10d also demonstrated higher activity, and their self-assembly properties enabled better cellular uptake. In the in vivo experiments in mice, 10d exhibited better antitumor effects and maintained its original body weight compared with the Cisplatin group, indicating stronger activity and fewer side effects. From the perspective of in vivo circulation, 10d had good stability and, due to its longer hydrophobic chain, showed a longer circulation time. Mechanistically, this class of drugs can induce high levels of ROS by depleting GSH, causing mitochondrial dysfunction in tumor cells, while still retaining Cisplatin’s DNA-binding function.

By coordinating with selenomethionine esters having different R groups, this Cisplatin analog exhibits different self-assembly behaviors, thereby modulating anticancer activity. This study has made a new contribution to the development of small-molecule self-assembly drugs [61].

#### 2.4.6. Pt-BA

**Figure 25:**
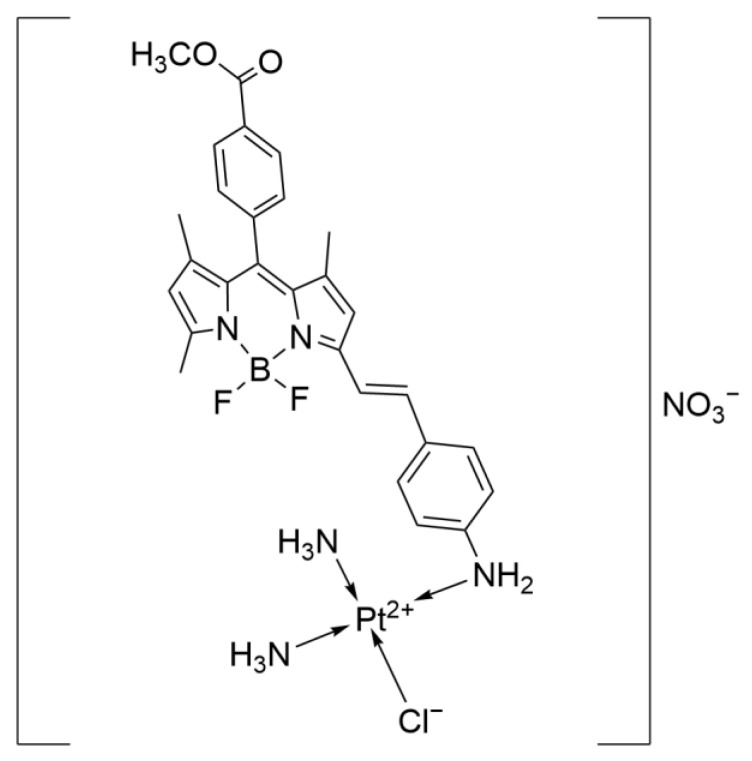
The structure of Pt-BA.

The Xulin team first combined the weak photosensitizer α-(4-amino)styryl-4,4-difluoro-4-bora-3a,4a-diaza-s-indacene (BA) with platinum to form a new complex, Pt-BA (Figure 25), which achieves efficient drug uptake and cell membrane damage through short-term light exposure.

Pt-BA avoids the dependence on continuous light exposure in traditional photodynamic therapy (PDT) and is actually more inclined to photodynamic activation chemotherapy (PACT). Compared with no light exposure, after 2 h of drug incubation, only 5 min of short-term light exposure can cause the drug to aggregate significantly in the cytoplasm. One hour after light exposure, the drug can even reach the cell nucleus. The mechanism of action is achieved by rapidly activating Pt-BA to induce the generation of ROS that destroy the cell membrane. In vitro experiments showed that the anticancer activity of Pt-BA increased significantly after short-term light exposure, with an IC_50_ of 9.7 μM in A549 cells, which is better than Cisplatin (IC_50_ = 10.1 μM).

This research result indicates that in actual lung cancer treatment, light can be targeted to the lesion site to artificially activate the “anticancer” switch in a specific area, eliminating tumors while avoiding damage to other normal cells. However, further experiments are still needed in the future to explore its specific target of action and optimize the absorption wavelength of the BA ligand to the longer near-infrared region (700–900 nm) to increase the penetration depth of the excitation light [62].

## 3. Discussion and Summary

Platinum-based complexes, having undergone over half a century of generational evolution, have consistently held a central position in lung cancer treatment. The first-generation Cisplatin established the foundation of chemotherapy through its DNA cross-linking mechanism. The second-generation Carboplatin and Nedaplatin reduced toxicity through structural modifications. The third-generation Oxaliplatin achieved a breakthrough in reversible neurotoxicity. Throughout these developments, the structural innovations of platinum complexes have always been guided by the dual objectives of “enhancing anticancer activity and reducing systemic toxicity.”

However, the limitations of traditional platinum complexes—dose-dependent toxicity, drug resistance, and insufficient targeting—remain urgent clinical challenges. In recent years, the development of novel platinum complexes has provided potential solutions to these bottlenecks. New platinum complexes (such as cycloplatin and fluoro-bipyridine analogs) and targeted delivery systems (inhaled SLIT Cisplatin, liposomal SPI-77) have shown different potentials in overcoming drug resistance and reducing systemic toxicity through structural modifications and precise delivery strategies.

Therefore, we further gathered additional literature on platinum-based complexes design and integrated this with existing knowledge. We propose that future research should focus the design of platinum complexes on the following aspects to better achieve the goal of “increased efficacy and reduced toxicity”: First, developing prodrugs (for example, Wang’s team oxidized the central platinum atom to Pt(IV), generating a six-coordinate, highly stable structure that exhibits superior chemical stability and aqueous solubility) [63]. Second, designing and optimizing the structures of multinuclear platinum complexes. Although BBR 3464, for example, is theoretically expected to bind strongly to negatively charged DNA because of its high positive charge, it has not shown a clear advantage over conventional drugs in practice. Third, incorporating non-classical ligands such as macrocyclic or targeting ligands. For instance, Philipp’s team appended a maleimide ligand and L-buthionine sulfoximine (BSO) to oxaliplatin: the maleimide extends the drug’s plasma half-life and reduces off-tumor toxicity, while BSO inhibits glutathione (GSH) synthesis, thereby overcoming GSH-mediated resistance to oxaliplatin [64]. Fourth, continued innovation in targeted delivery systems, such as refining nanoscale platinum and liposomal platinum formulations to achieve toxicity reduction. Fifth, creation of multifunctional platinum therapeutics. Building on the Pt-Exo concept, future platinum designs can be further conjugated with enzyme-linked inhibitors or immune modulators. Drawing on the Pt-BA strategy, platinum activation and release can be controlled by leveraging features of the tumor microenvironment or external stimuli, thereby enhancing tumor selectivity and lowering systemic toxicity. Moreover, platinum can be coupled with other metals (e.g., Se, Au) or natural agents (e.g., paclitaxel, camptothecin) to attack lung cancer from multiple angles, collectively driving platinum-based complexes from a paradigm of “broad-spectrum cytotoxicity” toward “precision therapy” [65].

## Figures and Tables

**Table 1:** This table compiles the structural characteristics and relevant marketing information of the three generations of marketed platinum-based complexes and Dicycloplatin.

Generation	Drug	Molecular Formula	Key Structural Features	Water Solubility Characteristics	First Launched Country	Launch Year
1st	Cisplatin	Pt(NH_3_)_2_Cl_2_	Cis-configuration, Cl^−^ ligands	Low (requires hydration therapy)	USA	1978
2nd	Carboplatin	Pt(NH_3_)_2_(C_2_O_4_)	Bidentate cyclobutane dicarboxylate ligand	High (l6× more soluble than Cisplatin, no hydration)	UK	1986
2nd Deriv.	Nedaplatin	Pt(NH_3_)_2_Cl(CH_3_COO)	Glycolic acid replaces two Cl^−^ in Cisplatin	High (~10× more soluble than Cisplatin, no hydration)	Japan	1995
3rd	Oxaliplatin	C_8_H_14_N_2_O_4_Pt	Cyclohexane ligand	Moderate (lower toxicity)	France	1980s
3rd Deriv.	Lobaplatin	C_9_H_18_N_2_O_3_Pt·3H_2_O	Cyclohexane ligand	Moderate (lower nephrotoxicity)	China	2005
Trial Phase	Dicycloplatin	C_12_H_20_N_2_O_8_Pt	Cyclobutane dicarboxylate ligand	Very high (low toxicity)	/	/

Tips: Dicycloplatin is currently in the clinical trial phase and has not yet been approved for marketing worldwide.

**Table 2:** This table compiles the therapeutic scope and adverse reactions of the three generations of complexes.

Drug	Indication	Major Toxicities	Target Patient Population
Cisplatin	NSCLC, SCLC	Nephrotoxicity, nausea/vomiting, ototoxicity	Patients needing rapid symptom relief in extensive-stage SCLC
Carboplatin	NSCLC (patients with renal insufficiency)	Myelosuppression, thrombocytopenia	Patients with renal insufficiency
Nedaplatin	SCLC	Myelosuppression, neutropenia, thrombocytopenia	Patients requiring low nephrotoxicity regimens
Oxaliplatin	NSCLC (neuropathy-intolerant patients)	Reversible peripheral neuropathy	Patients intolerant to Cisplatin toxicity
Lobaplatin	NSCLC (Chinese patients)	Myelosuppression, thrombocytopenia	Patients requiring low gastrointestinal toxicity regimens
